# Barriers to Women's Leadership in Nursing: A Rapid Evidence Assessment and Proposal for an Integrated Theoretical Framework

**DOI:** 10.1111/jan.70570

**Published:** 2026-04-10

**Authors:** Chiara Palazzo, Vincenza Giordano, Andrea Chirico, Teresa Rea, Assunta Guillari

**Affiliations:** ^1^ Department of Biomedicine and Prevention University of Rome ‘Tor Vergata’ Rome Italy; ^2^ Department of Public Health University of Naples ‘Federico II’ Naples Italy; ^3^ Department of Social and Developmental Psychology University of Rome ‘Sapienza’ Rome Italy; ^4^ Department of Translational Medical Sciences University of Naples ‘Federico II’ Naples Italy

**Keywords:** barriers, career progression, gender bias, gender discrimination, gender gap, glass ceiling, leadership, nursing, organisational and cultural factors, sex discrimination

## Abstract

**Aim(s):**

This review analyses and synthesises the available evidence on the barriers limiting women's career advancement in nursing. It aims to identify effective interventions to promote gender equity in healthcare leadership through an integrated framework informed by Social Role Theory, Ambivalent Sexism Theory and Theory of Planned Behaviour.

**Design:**

Rapid Evidence Assessment.

**Data Sources:**

The search was conducted on the databases: PubMed, CINAHL Complete, MEDLINE, ApaPsycArticles, ApaPsycInfo between March and May 2025.

**Methods:**

The Population–Exposure–Outcome framework guided the formulation of the research question. Two reviewers independently conducted screening, quality appraisal and data extraction.

**Results:**

Eight studies with heterogeneous research designs were included: four literature reviews, three quantitative studies and one qualitative study. The analysis identified four themes: (1) gender pay gap; (2) gender stereotypes and cultural expectations; (3) systemic barriers: organisational structures and institutional culture; (4) organisational strategies for gender equity and female leadership.

**Conclusion:**

Career advancement for female nurses is limited by systemic and internalised constraints that reinforce vertical segregation. Multilevel interventions are needed to dismantle structural inequalities and reshape gender norms within nursing leadership.

**Implications for the Profession and/or Patient Care:**

Targeted reforms, including transparent promotion criteria, structured mentoring, bias awareness training and inclusive organisational policies, are essential to promote leadership equity, improve workplace justice and foster a sustainable health system.

**Impact:**

This study addresses the persistent gender gap in nursing leadership, identifying systemic, cultural and individual barriers to women's career progression. Through a Rapid Evidence Assessment, it proposes a new multilevel theoretical framework. The results support policies and interventions to promote gender equity in nursing leadership.

**Reporting Methods:**

This review adheres to the Preferred Reporting Items for Systematic Reviews and Meta‐Analyses guidelines for reporting.

**Patient or Public Contribution:**

None.

## What Does This Paper Contribute to the Wider Global Clinical Community?

- Provide a novel theoretical integration by combining Social Role Theory, Ambivalent Sexism Theory and Theory of Planned Behaviour to interpret gendered leadership disparities in nursing, offering a multidimensional framework applicable across healthcare systems.
- Identifies system‐wide barriers, including implicit bias, pay inequity and lack of institutional support, that hinder women nurses' advancement to leadership roles, highlighting the need for structural and cultural reforms in healthcare organisations globally.
- Supports evidence‐informed policy development, offering actionable recommendations such as mentoring, flexible career pathways and early education on gender equity to improve female representation in leadership within the nursing profession internationally.

## 1 Introduction

Gender equality in career trajectories remains one of the most complex and pressing challenges in contemporary society, spanning social, political and economic dimensions. Although significant progress has been made regarding rights and equal opportunities, marked disparities persist in women's representation at senior organisational levels, in wage structures and in access to leadership positions (World Health Organization 2021). The so‐called ‘gender leadership gap’ is not only an indicator of systemic inequity but also a barrier to sustainable development, economic growth and social innovation.

Gender, understood as a social construct referring to culturally defined roles, responsibilities, attributes and rights associated with being male or female within a specific context (Heise et al. 2019), exerts a structural influence and constitutes a significant determinant of workplace discrimination (Cottingham 2019). To understand gender dynamics in professional settings more deeply, it is crucial to conceptually distinguish three levels of gender‐related asymmetry: stereotypes, prejudice and discrimination. Gender stereotypes are generalised and reductive beliefs that attribute specific characteristics to men and women based on gender membership (Heilman 2001). Prejudice is an evaluative attitude, either positive or negative, resulting from such stereotypes (Eagly and Mladinic 1994). Finally, discrimination refers to the behavioural manifestation of prejudice, translating into actions or decisions that systematically penalise one gender over the other (Fiske 2000). These three constructs lie along a continuum from cognitive representation to behavioural expression, and they represent a set of mechanisms that, within the workplace, can negatively influence the assessment of skills, qualifications and role suitability, limiting women's access to leadership positions and impeding their career advancement (Newman 2014). An effect of stereotypes and prejudices, understood as deeply rooted cognitive and attitudinal beliefs, is the emergence of biases. Biases can be both implicit and explicit, referring to systematic cognitive or attitudinal inclinations that shape perceptions and decisions toward individuals or groups. Explicit bias involves conscious and intentional attitudes or judgements, while implicit bias operates unconsciously and automatically, influencing behaviour even in the absence of deliberate intent. Biases, originating from stereotypes and prejudices, can result in discriminatory practices in professional and organisational contexts (FitzGerald and Hurst 2017; Llorens et al. 2021).

To date, the gender leadership gap remains a global issue that cuts across all professional domains (Chisholm‐Burns et al. 2017), including healthcare settings. Several studies have explored this phenomenon in fields such as medicine (Harris et al. 2021), clinical psychology (Reynolds et al. 2022), academic medicine (Odei et al. 2021), as well as in specific specialties such as anaesthesiology (Schwartz et al. 2021) and surgery (Tirumalai et al. 2021).

Globally, women comprise approximately 70% of the workforce in the health and social care sectors, providing care for more than 5 million people and representing about 234 million workers in total. The highest concentration of female workers is found in nursing and midwifery, where they represent up to 90% of the workforce (Smith and Sinkford 2022). In Europe, according to the 2019 Eurostat Report, women constitute 78% of the 4.5 million nurses and midwives, a proportion consistent with the Italian figure of 76% (D'Artibale et al. 2025). However, despite this numerical predominance, women remain significantly underrepresented in decision‐making roles, occupying only 25% of leadership positions in the health sector (Kark and Buengeler 2024; World Health Organization 2019). This disparity is even more pronounced in low and middle‐income countries, where women from disadvantaged socioeconomic backgrounds face multiple, intersectional and systemic barriers (Global Health 50/50 2020; World Health Organization 2021). The COVID‐19 pandemic has further exacerbated these inequalities, affecting women significantly more severely in terms of both employment and career progression (Beugelsdijk et al. 2025; Smith and Sinkford 2022; World Health Organization 2019).

As highlighted in a joint report by the World Health Organization (WHO) and the International Labour Organization (ILO), women in the healthcare sector receive on average 20% less pay than men for equal roles and responsibilities (International Labour Organization 2022; World Health Organization 2022). In response to these persistent inequalities, the WHO established the Gender Equity Hub (GEH) in 2017 during the *Fifth Global Forum on Human Resources for Health*. The GEH represents a strategic platform aimed at promoting gender equity in the health and social care workforce through the development of disaggregated data, inclusive policies and transformative practices. Among its key outputs, the report ‘Delivered by Women, Led by Men’ emphasised the urgency of addressing systemic gender biases and disparities that limit women's access to leadership positions. These initiatives fall within the broader scope of the Sustainable Development Goals (SDGs), particularly those related to gender equity (SDG 5) and decent work (SDG 8) (World Health Organization 2019, 2021). In parallel, several relevant initiatives have emerged, such as the *Women's Wellness through Equity and Leadership* (WEL), promoted by international medical organisations to facilitate the creation of equitable work environments and women's leadership development (Kelly et al. 2021). Building on this context, the term ‘*leadership*’ refers primarily to managerial and organisational leadership, defined as access to formal decision‐making, executive and strategic positions within healthcare systems. This is conceptually distinct from clinical leadership, which is grounded in direct care, professional expertise and practice‐based influence (Stanley and Stanley 2018; Cardiff et al. 2018).

## 2 The Review

To understand women's access to leadership in nursing, there exist psychological, cultural and organisational barriers that systematically limit career progression. Theoretical frameworks have been developed to explain these mechanisms, linking gender norms, attitudes and structural constraints. Against this theoretical background, this review proposes an integrated interpretative framework combining Social Role Theory, Ambivalent Sexism Theory and the Theory of Planned Behaviour to synthesise evidence on leadership barriers in the nursing context.

Social Role Theory (SRT) (Eagly and Wood 1999) represents a fundamental starting point by emphasising the sexual division of labour, generating an unequal distribution of roles and careers between genders, with a greater concentration of women in some professions and men in others. It is widely believed that men and women possess certain qualities and traits that predispose them to particular types of roles and responsibilities (Eagly and Steffen 1984). These social representations shape perceptions of professional skills and aspirations, reinforcing gender segregation and limiting women's advancement into leadership roles.

Within this cultural background, Ambivalent Sexism Theory (AST) (Glick and Fiske 2018) offers further insight into how gender‐related attitudes can manifest themselves in two distinct yet complementary forms of sexism: a hostile one, expressed in openly discriminatory attitudes and a benevolent one, manifested in apparently positive paternalistic ways that reinforce women's subordination. Both forms of sexism, often internalised even by women themselves, feed the idea of a presumed natural inclination of women to caring roles, contributing to the reproduction of gender stereotypes and invisible obstacles to access to leadership positions.

At the individual level, the Theory of Planned Behaviour (TPB) (Ajzen 1991) allows us to understand how personal attitudes toward behaviour, perceived subjective norms and a sense of behavioural control influence behavioural intentions. This theory makes it possible to interpret how social expectations, perceptions of ineffectiveness and the internalisation of limiting role models may reduce women's propensity to apply for leadership roles even when they possess the required appropriate skills.

Completing this theoretical framework are the organisational concepts of vertical segregation, glass ceiling and sticky floor. ‘*Vertical segregation*’ describes a hierarchical division in which, despite working in the same profession, men occupy more responsible and better‐paid roles than women (Hannawi and Al Salmi 2018; Jarman et al. 1999). The ‘*glass ceiling*’ is a metaphor describing the invisible but systematic barriers that prevent women from reaching leadership or senior positions in their careers, despite having the necessary qualifications and skills (Kapoor et al. 2021). In contrast to men who experience a ‘*glass escalator*’ effect, a social and organisational phenomenon that describes a faster rise to senior roles or leadership positions in a profession that is more represented by women, such as nursing (Chisholm‐Burns et al. 2017; Newman 2014; Turkmen and Bacaksiz 2021; Williams 2013). The ‘*sticky floor*’, on the other hand, refers to those cultural and structural dynamics that keep women confined to the lower levels of organisational hierarchies, preventing them from accessing the highest‐level roles (Srivastava and Nalawade 2023).

Nursing is considered one of the most strongly gendered professions globally (Australian College of Nursing 2019). Historically perceived as a female vocation due to its caring and relational nature, nursing has been socially and culturally shaped to align with traditional female roles. Although men have always contributed to nursing and care work in various settings, their presence has often been underrepresented or redefined through gendered expectations, reinforcing the perception of nursing as a predominantly female profession (Alghamdi et al. 2018). This social construction is rooted in cultural legacies that associate the nursing role with low occupational status, considered a natural extension of domestic activities traditionally performed by women as wives and mothers (Clayton‐Hathway et al. 2020; Royal College of Nursing 2020). This gender‐based division of labour has gradually channelled men into specialised and leadership roles (Evans 2004). These advantages appear to be linked to power dynamics and the valorisations of the agentic traits typically attributed to men that facilitate their professional rise even in female‐dominated contexts (Brown and Jones 2004). Comparative studies confirm that the average time taken to attain management roles in nursing is significantly shorter for men than for women, 8.4 years vs. 17.9 years (Lane and Piercy 2003). In contrast, minority male status is not a barrier to career advancement in the same way as it is for women in male‐dominated professional fields (McMurry 2011).

## 3 Aims

The gender gap in nursing leadership, therefore, is a structural and persistent challenge. Despite growing academic interest in this topic, the literature still appears fragmented and often focused on individual factors, without a systemic and theoretically grounded framework of barriers and corrective strategies.

Consequently, this review responds to this gap by providing a systematic analysis of the phenomenon and the barriers impeding women nurses' career progression, through the lens of a structured theoretical framework as an interpretative approach to synthesise and contextualise the findings. Given the current global emphasis on gender equity in healthcare leadership and workforce sustainability, this study offers timely evidence for multilevel interventions that promote women's advancement in nursing leadership, supported by an integrated framework.

## 4 Method

### 4.1 Design

A Rapid Evidence Assessment (REA) was conducted following established methodological guidance for rapid evidence synthesis (Watt et al. 2008; Khangura et al. 2012; Varker et al. 2015). In line with Varker et al. (2015), this REA retained core systematic review principles, including explicit eligibility criteria, reproducible search strategy, structured screening and quality appraisal, while applying pragmatic limits to scope and timeframe.

### 4.2 Search Methods

The research question for this review was defined following the PEO method (Bettany‐Saltikov and McSherry 2024). ‘What individual, cultural and organisational factors influence the career progression of women in the nursing profession?’.

Specifically:

*Population*: female nurses.

*Exposure*: individual, cultural and organisational factors.

*Outcome*: career progression and access to leadership positions.

The literature search was conducted using the following electronic databases: PubMed, CINAHL Complete, MEDLINE, ApaPsycArticles and ApaPsycInfo. The search was carried out between March and May 2025. The main search terms used were *gender bias*, *gender gap*, *gender discrimination*, *career progression*, *organisational and cultural factors*, *sex discrimination*, *leadership*, *glass ceiling*, *nursing* and *barriers*. Search strings were constructed using Boolean operators ‘OR’ and ‘AND’, combining the terms to optimise the sensitivity and specificity of the literature search.

In line with the REA approach, quantitative, qualitative and mixed‐method primary studies were included, as well as literature reviews with the aim of mapping the state of the art more broadly and possibly supplementing the primary data with already synthesised evidence. This inclusion strategy was methodologically justified by the interpretative and theory‐building aim of the review. Integrating higher‐order syntheses with context‐specific primary evidence enabled a comprehensive conceptual mapping of structural, cultural and individual barriers to leadership progression, rather than the estimation of aggregated effects. The review therefore prioritised conceptual integration over statistical aggregation, in line with rapid evidence assessment guidance (Varker et al. 2015). Grey literature was not included as part of the pragmatic scope restrictions applied in this REA, consistent with rapid evidence synthesis approaches that prioritise feasibility within defined timeframes (Varker et al. 2015). As this study involved the synthesis of previously published research, no ethical approval was required.

### 4.3 Inclusion and Exclusion Criteria

The following inclusion and exclusion criteria were established for the literature search.

*Inclusion criteria*: (a) primary studies; (b) original studies; (c) literature reviews; (d) articles that comprehensively reported on barriers to career progression of women nurses in healthcare settings; (e) articles in English; (f) peer‐reviewed journals; and (g) articles published from 2015 to 2025.

*Exclusion criteria*: (a) guidelines; (b) dissertations, oral presentations, methodological or theoretical descriptions or individual clinical cases; (c) studies that did not comprehensively investigate barriers to career progression of women nurses in healthcare settings; (d) articles written in languages other than English; and (e) articles published before 2015.

### 4.4 Search Outcome

The study selection process initially identified 8401 articles, of which 6692 came from PubMed and 1709 from the CINAHL Complete, MEDLINE, APA PsycArticles and APA PsycInfo databases. No registers were used. All records were uploaded and managed through Rayyan—Intelligent Systematic Review software to facilitate the selection process and reduce the risk of bias. Initial screening by title was performed, eliminating duplicates. This phase was conducted independently by two reviewers (C.P. and A.G.); any conflicts were resolved through consensus discussion. At the end, 28 articles were selected for full‐text reading. During this phase, 20 articles were excluded for the following reasons: 15 did not meet the inclusion criteria, and 5 were primary studies already included in the selected reviews, and therefore excluded to avoid duplication of data. Consequently, 8 studies were included in the final review.

To minimise the risk of overlap and potential ‘double counting’, we cross‐checked the reference lists of included reviews against retained primary studies during full‐text screening. Primary studies substantially represented within included reviews were excluded unless they provided additional contextual, methodological or temporally updated evidence. The synthesis followed a segregated analytic approach, whereby review‐level findings and primary study findings were analysed independently prior to triangulation. No quantitative pooling, cumulative frequency counting or weighting of evidence across study types was undertaken. Integration occurred at the interpretative level only, thereby preventing duplication of empirical contribution.

**FIGURE 1:**
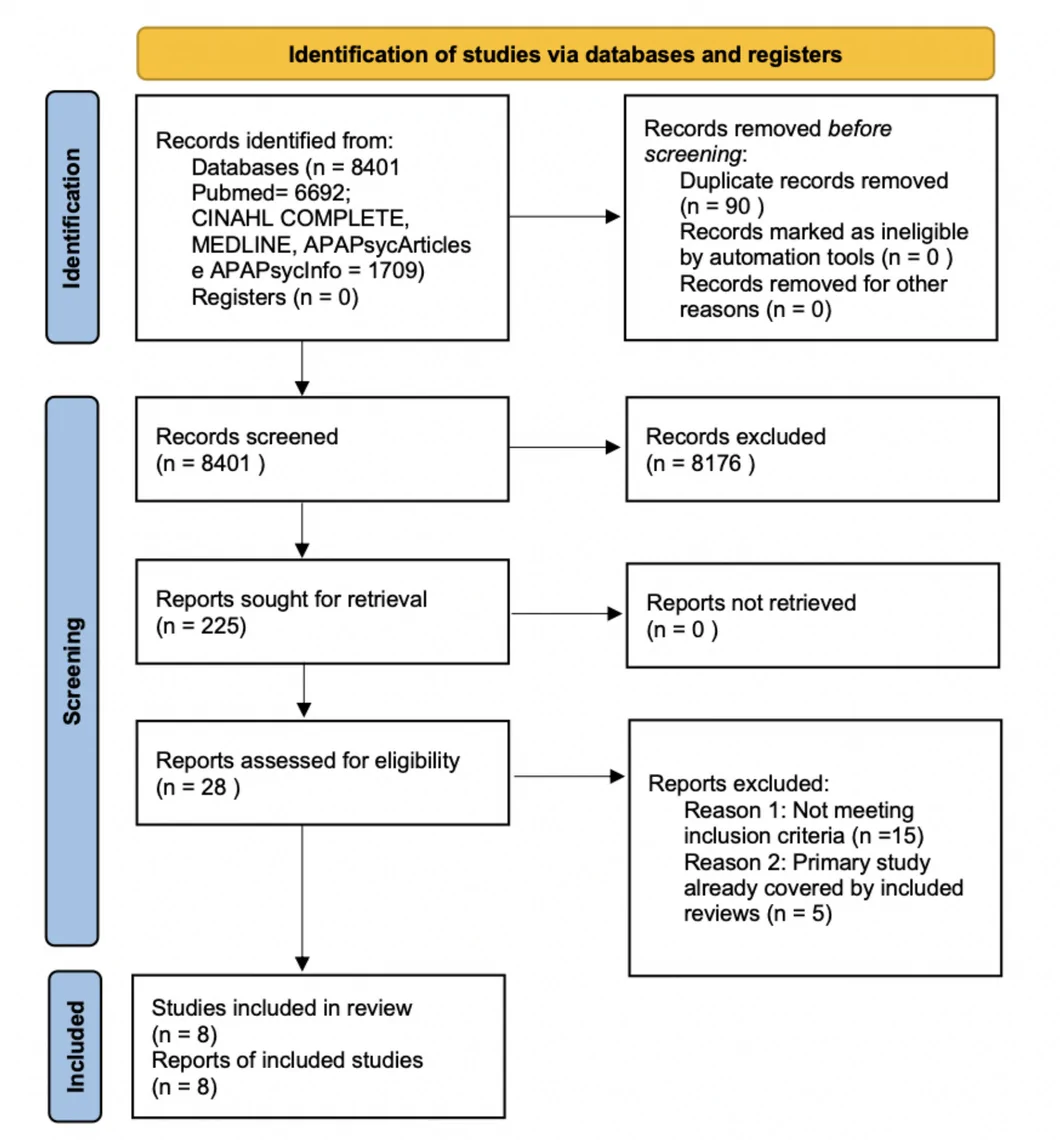
PRISMA diagram flow (Page et al. 2021).

The processes of identification, screening, eligibility and inclusion were systematically documented and are presented graphically using a PRISMA 2020 flow diagram (Figure 1), adapted to the methodological context of a REA in accordance with PRISMA guidelines (Page et al. 2021). Reporting of this REA was informed by the PRISMA 2020 Statement (EQUATOR Network), with methodological adaptations consistent with established REA guidance (Varker et al. 2015). A PRISMA checklist adapted for REA reporting is provided as Supporting Information.

### 4.5 Quality Appraisal

The methodological quality and risk of bias of the included studies were assessed following the principles of REA and integrative reviews, which emphasise methodological transparency while maintaining analytical efficiency (Whittemore and Knafl 2005; Varker et al. 2015). Given the methodological diversity of the included studies, validated design‐appropriate tools were used. Qualitative studies and literature reviews were appraised using the QuADS tool (Quality Appraisal for Diverse Studies), developed and validated by Harrison et al. (2021), which is suitable for synthesising evidence from diverse methodological approaches. A maximum score of 39 was possible, and predefined thresholds were used to categorise as high quality (Harrison et al. 2021; Heilman 2001; Heise et al. 2019; International Council of Nurses 2023; International Labour Organization 2022; James et al. 2025; Jarman et al. 1999; Joanna Briggs Institute 2022; Kapoor et al. 2021; Kark and Buengeler 2024), moderate quality (FitzGerald and Hurst 2017; Frantzen and Fetters 2016; Gauci et al. 2023; Glick and Fiske 2018; Global Health 50/50 2020; Goldsmith et al. 2007; Granikov et al. 2022; Greene et al. 2017; Hannawi and Al Salmi 2018; Harris et al. 2021) or low quality (< 20). Quantitative cross‐sectional studies were evaluated using the JBI Checklist for Analytical Cross‐Sectional Studies (Joanna Briggs Institute 2022), with scores ranging from 0 to 8 and categorised as high (Beugelsdijk et al. 2025; Brown and Jones 2004), moderate (Bandura 1977; Bettany‐Saltikov and McSherry 2024) or low quality (< 5). All appraisals were independently conducted by two reviewers, with disagreements resolved through discussion, ensuring inter‐rater reliability. The risk of bias was considered directly proportional to the assessed quality. For quantitative studies, potential biases included sample selection, lack of adjustment for confounders and reliance on self‐reported data. For qualitative and review studies, methodological limitations included insufficient theoretical justification, limited stakeholder involvement and reduced analytic transparency. These factors were used to determine the level of confidence in the evidence: findings from studies with high methodological quality and low risk of bias were considered robust, while those with moderate or low quality were interpreted with caution and contextualised through data triangulation. Detailed quality scores and risk of appraisals are reported in Tables S1 (QuADS) and S2 (JBI).

### 4.6 Data Extraction

To ensure a clear, systematic and coherent synthesis of the included studies, data were extracted and organised into three distinct thematic tables based on study design: systematic and integrative review (Table S3), quantitative studies (Table S4) and qualitative studies (Table S5). Each table includes the following elements, consistently adapted from validated frameworks for mixed‐methods systematic reviews: author(s) and publication year, study objective/focus, country or setting, sample characteristics, study design, type of analysis (for qualitative studies), main findings, conclusions and implications for practice. This structured tabular format was chosen to facilitate both intra‐method comparison (among similar study types) and cross‐method integration across different methodologies (Frantzen and Fetters 2016; Lizarondo et al. 2025). The data extraction process was informed by methodological guidance for mixed studies reviews, especially the convergent integrated approach, which emphasises preserving the integrity of findings from each method before integration. This method supports transparent synthesis and enhances interpretability by aligning extracted data to common analytical domains across study types (Granikov et al. 2022; Stern et al. 2020). The data extraction template was pilot tested on a subset of included studies and iteratively refined to ensure consistency and reliability across reviewers.

Where appropriate, qualitative and quantitative findings were juxtaposed to enhance thematic coherence and support integrative interpretations (Goldsmith et al. 2007).

### 4.7 Synthesis

The data were synthesised using a segregated narrative synthesis model, which involved the separate analysis of findings from quantitative, qualitative studies and literature reviews.

Subsequently, the results were triangulated to identify thematic convergences and divergences. This strategy made it possible to preserve the methodological integrity of the different approaches while ensuring an integrated view of the phenomena analysed.

## 5 Results

This review included eight articles published between 2015 and 2025, selected based on criteria of originality, relevance and methodological rigour. The studies are divided into: Four literature reviews (Aspinall et al. 2023; Gauci et al. 2023; Pincha Baduge et al. 2024; Saville et al. 2024), three quantitative studies (Doleman et al. 2024; Greene et al. 2017; Muench and Dietrich 2019), one qualitative study (Mousa et al. 2023). The limited number of included studies, together with the heterogeneity of their designs, affects the overall depth and comparability of the available evidence. However, this scope is consistent with the methodological principles of REA (Varker et al. 2015), which prioritises analytical rigour, timeliness and the avoidance of redundant or overlapping data. The focused inclusion of only the most methodologically robust and conceptually relevant studies enhances the validity, transparency and interpretability of the findings.

The methodological quality of the included studies was appraised using the QuADS tool for reviews and qualitative research and the JBI Critical Appraisal Checklist for quantitative designs (Tables S1 and S2). Overall, studies demonstrated moderate‐to‐high methodological quality. Review papers showed strong theoretical grounding and well‐defined research aims, though analytic justification and discussion of limitations varied (Aspinall et al. 2023; Gauci et al. 2023; Pincha Baduge et al. 2024; Saville et al. 2024). The qualitative study (Mousa et al. 2023) was assessed as high quality, showing clear methodological coherence, appropriate use of grounded theory and rich interpretive depth supported by participant quotations. Quantitative studies achieved high scores in design transparency and validity (Doleman et al. 2024; Greene et al. 2017), with one rated moderate due to limited control of confounding factors (Muench and Dietrich 2019). These findings support the robustness of the included evidence base.

The studies were conducted across a range of geographical settings, including Australia, New Zealand, Germany, the United States, India and Kenya, reflecting systemic, cultural and organisational diversity. These differences influence both the nature and intensity of gender inequalities in nursing leadership. However, despite the heterogeneity of contexts, a thematic convergence emerged: structural, cultural and economic barriers to women's progression in the nursing profession are pervasive and systemic.

The evidence collected was organised into four macro‐themes: (1) gender pay gap, (2) gender stereotypes and cultural expectations, (3) systemic barriers: organisational structures and institutional culture and (4) organisational strategies for gender equity and women's leadership.

For each theme, evidence was compared between quantitative, qualitative and review studies. Where possible, interpretative convergences (transversal presence of the gender pay gap) and methodological divergences (variability in the measurement of barriers) were highlighted. This triangulation enhanced the internal coherence of the synthesis and the validity of the inferences, supporting a critical and multidimensional interpretation of the phenomenon studied.

### 5.1 Gender Pay Gap

The gender pay gap emerges as one of the most documented and persistent barriers to women's career progression in the nursing profession. The quantitative studies included show significant salary differences against women that are not justified by individual, professional or contractual factors. In Germany, Muench and Dietrich (2019) report a 9.3% wage gap in favour of men, which persists even in the presence of equivalent working conditions or in greater professional experience. In Australia, Doleman et al. (2024) note an average wage gap of AUD 5700 against female nurses. The gap is already evident in the first 6 months after graduation (4%) and increases to 13% after 3 years of employment. This trend suggests a gradual erosion of economic opportunities for women over time, with less valuation of their professional contributions. The authors attribute part of this disparity to factors that cannot be explained by observable variables, pointing to the influence of implicit structural and gender mechanisms, such as stereotypes in career paths, assignment to less economically valued roles and limited access to internal advancement.

One of the possible justifications identified is men's greater availability to work shifts less compatible with family responsibilities (such as night and weekend shifts), compared to women, who are more often involved in caregiving duties.

In the United States, Greene et al. (2017) show a significant salary gap among nurse practitioners (NPs), which remains even after accounting for age, education, experience, clinical specialty and geographic location. The residual gap exceeds $12,000 annually in favour of men. Inequality is already present early in the career and tends to widen over time, highlighting systemic and persistent mechanisms of discrepancy in wage determination. The evidence from the quantitative studies is further supported by the included reviews and qualitative study. These works recognise the gender pay gap not only as an indicator of economic inequality, but as a systemic and structural manifestation of persistent disparities in the nursing profession (Aspinall et al. 2023; Gauci et al. 2023; Mousa et al. 2023).

### 5.2 Gender Stereotypes and Cultural Expectations

Gender stereotypes and cultural expectations associated with women's roles emerge as one of the most entrenched and pervasive barriers to the career progression of women nurses. Traditional representations of nursing leadership remain strongly anchored in culturally associated masculine traits such as assertiveness, rationality, technique and leadership orientation (Aspinall et al. 2023; Pincha Baduge et al. 2024; Saville et al. 2024). Conversely, women are identified with relational and caring characteristics, such as empathy, communication and emotional availability, which, while valued in the clinical role, are often incompatible with decision‐making roles.

These representations influence not only external judgement but also professional behaviours and self‐perceptions. As noted by Gauci et al. (2023), men and women in coordinating positions tend to internalise stereotypical roles: the former are encouraged to demonstrate authority and technical leadership, while the latter focus on the relational dimension, thus generating implicit constraints on the autonomy and visibility of female leadership. Such dynamics contribute to women's lower confidence in their abilities, despite possessing adequate professional skills and may limit their propensity to assume strategic or exposed roles.

This perceived inequality is further reflected in Mousa et al. (2023) qualitative study, in which women leaders report the need to adapt their leadership style to male models to be recognised, partly giving up professional authenticity. Barriers manifest themselves not only as explicit forms of exclusion but through normalisation processes that tend to make forms of subordination invisible. Finally, a further gender paradox emerges: although men represent a minority in the nursing profession, they seem to benefit from the ‘glass elevator’ that facilitates their advancement into leadership positions, thanks to greater institutional recognition and easier legitimisation of male authority (Gauci et al. 2023).

### 5.3 Systemic Barriers: Organisational Structures and Institutional Culture

Gender inequalities in nurses' career progression can also be traced by the interaction of two closely intertwined levels: on the one hand, structural and operational barriers, which are visible and linked to organisational processes; on the other hand, a silent, but equally decisive, institutional culture that acts through values, implicit norms and lack of institutional awareness. Structural barriers include concrete and formalised mechanisms that regulate access to leadership: lack of transparency in promotion criteria, poor access to mentoring programmes, absence of leadership training paths, organisational rigidity in workloads and limited support for work‐life balance (Gauci et al. 2023; Pincha Baduge et al. 2024; Saville et al. 2024). Though visible, these obstacles are difficult to overcome without systemic organisational reform.

In parallel, institutional culture acts as an invisible barrier, operating through the lack of a shared vision on gender equity, the absence of a culture of gender diversity and the reproduction of leadership patterns implicitly associated with masculinity (Aspinall et al. 2023). As pointed out by Aspinall et al. (2023), an effective analysis of these dynamics requires an intersectional approach that considers how gender, ethnicity, class and occupational status may interact in generating overlapping forms of disadvantage. Failing to address these intersections may render inclusion policies ineffective, particularly for women who occupy multiple marginalised identities. In contexts such as India and Kenya, barriers are further exacerbated by rigid social norms, wage disparities and a lack of work flexibility, which limit the scope of remedial initiatives (Saville et al. 2024). In summary, systemic barriers to women's leadership in nursing stem from the intersection of concrete organisational barriers and normalised institutional practices that reproduce not only gendered but also social and cultural power hierarchies.

### 5.4 Organisational Strategies for Gender Equity and Women's Leadership

Although the focus of the review was the identification of barriers to career progression for women nurses, several studies also proposed operational strategies that deserve to be considered as part of the interpretive framework. In this sense, the fourth theme brings together the main proposals that emerged, aimed at addressing the inequalities highlighted in the previous themes. Some of these strategies stem directly from the barriers identified: what is missing or hindering, such as work flexibility, mentoring or leadership training, is also recognised as a priority area for intervention.

The identification of transformative organisational interventions is based on the recognition of two central theoretical concepts: gender stereotypes and implicit biases. On one hand, traditional stereotypes tend to portray men as agents, assertive and command‐oriented, while women are associated with relational and caring roles, limiting their perceived legitimacy in decision‐making contexts (Mousa et al. 2023; Pincha Baduge et al. 2024; Saville et al. 2024). On other hand, gender biases, often unreported, influence access to professional opportunities and merit recognition (Aspinall et al. 2023; Gauci et al. 2023). Response formal mentoring programmes have been widely recommended as one of the most effective interventions.

These strategies facilitate women's access to influential professional networks, foster the development of strategic skills and contribute to the strengthening of professional identity as leaders. At the same time, the importance of explicit recognition of gender bias in organisations and the activation of measures to ensure equal access to resources, benefits and career paths, calibrated to the real needs of female professionals, is emphasised (Mousa et al. 2023).

Comprehensive organisational policies, including transparent promotion criteria, equitable access to training, parenting support and work flexibility, are often referred to. However, as noted by Saville et al. (2024), the impact of these instruments has still been little investigated in terms of their effectiveness, especially in reducing structural inequalities. Without cultural transformation, these measures risk remaining isolated and ineffective.

Finally, several studies call for the need to adopt an intersectional approach, which considers how gender intersects with ethnicity, class and occupational status. This approach is considered crucial to prevent equity strategies from reproducing neutral or partial models (Aspinall et al. 2023; Pincha Baduge et al. 2024; Saville et al. 2024). Overall, overcoming inequities in nursing leadership requires coherent, multilevel interventions that combine individual empowerment with a structural reform of organisational practices and cultural norms.

## 6 Discussion

This review highlighted how the career progression of female nurses is limited by an interconnected system of barriers that operate at a cultural, psychological and structural level. Considering the multidimensional nature of gender inequality in nursing leadership is essential, as structural, cultural and psychological factors interact to sustain systemic barriers. Recognising this complexity helps frame the findings within a comprehensive interpretative lens and supports the need for multilevel strategies that address both organisational systems and individual perceptions. The four thematic areas identified intersect, revealing a systemic phenomenon in which career opportunities for female nurses are conditioned by dynamics that go far beyond the individual dimension.

The results were interpreted using an integrated theoretical framework that aimed to explain the findings at multiple levels, providing a comprehensive explanatory lens. The integration of Social Role Theory, Ambivalent Sexism Theory and the Theory of Planned Behaviour was applied retrospectively to interpret the synthesised evidence. Each theory offered a complementary perspective to understand how structural, cultural and individual mechanisms interact to sustain gender inequalities in nursing leadership.

Within this interpretative lens, the use of the TPB (Ajzen 1991) made it possible to interpret inequalities not only as external constraints but also as internalised processes: perceived social norms, cultural stereotypes and low confidence in one's abilities contribute to reducing women's motivation and intention to access top positions. From this perspective, leadership is not simply denied, but often self‐limited by a socially constructed sense of illegitimacy. Quantitative evidence on the gender pay gap (Doleman et al. 2024; Greene et al. 2017; Muench and Dietrich 2019) is also consistent with the theoretical framework adopted. According to TPB, the observed gender‐based wage disparities likely influence perceived behavioural control and subjective norms, thereby reducing intention to seek leadership roles. This aligns with evidence suggesting that awareness of unequal pay contributes to negative attitudes toward leadership, weakened perceived self‐efficacy and lower motivation to apply for senior roles.

This stereotypical attribution mechanism fuels a reduction in the perception of self‐efficacy (Bandura 1977), contributing to an implicit renunciation of leadership, which is fully consistent with the ‘M’ for motivation in the AMO model used in Pincha Baduage's review (2024). The motivation to access leadership is strongly compromised by cultural and relational factors: reduced self‐efficacy, the internalisation of subordinate roles, difficulties in reconciling work and life and the lack of formal support undermine the sense of legitimacy and the drive for professional advancement.

These dynamics also find a solid foundation in Social Role Theory (Eagly and Wood 1999) and Ambivalent Sexism Theory (Glick and Fiske 2018), which highlight how leadership is still socially coded as a male function, associated with directive, technical and individualistic traits. The qualitative study (Mousa et al. 2023) and the reviews analysed (Gauci et al. 2023; Pincha Baduge et al. 2024) show that female nurses, on the contrary, are frequently perceived and perceive themselves as possessing relational and caring skills, which are considered less compatible with top management roles.

These attributions reflect the persistence of rigid gender stereotypes (Heilman 2001), which contribute to defining in a normative way what is considered appropriate for women and men, consolidating the definition of gender discrimination (Fiske 2000).

However, the barriers to female leadership are not limited to internalised processes. At the structural level, they are supported and amplified by structural and organisational obstacles, which act systemically to limit women's access to top positions. Several studies included (Aspinall et al. 2023; Gauci et al. 2023; Saville et al. 2024) highlight how healthcare contexts are characterised by opaque promotional pathways, poorly formalised advancement criteria and limited access to structured mentoring programmes. In many contexts, healthcare organisations are hierarchical and uninclusive environments, where the apparent neutrality of procedures masks systematically discriminatory practices. These obstacles are reinforced in low‐ and middle‐income contexts, where patriarchal cultural norms, combined with a lack of resources and infrastructure, amplify pre‐existing inequalities (Saville et al. 2024). This pattern has also been observed across different healthcare systems globally, where gendered organisational structures continue to reproduce inequality despite policy‐level commitments to equity (Mucheru et al. 2024; James et al. 2025).

Most of the included studies originated from high‐income countries, reflecting the current global imbalance in research on gender and nursing leadership. Although this trend mirrors the distribution of academic production, it also highlights the need for a more balanced and critically inclusive representation of studies from low‐ and middle‐income countries. The interpretation of these findings should also consider the influence of cultural and institutional contexts, as gender norms, organisational hierarchies and societal expectations vary across countries and may shape how barriers to women's leadership are perceived and reported.

These contextual and geographical gaps also partly explain the limited and heterogeneous evidence base identified in this review. Such scope is consistent with the methodological intent of the REA approach, which emphasises analytical rigour, efficiency and the prevention of overlap between primary and secondary sources by focusing on methodologically robust and conceptually relevant studies.

The dynamics of vertical segregation, the ‘*glass ceiling*’, and the presence of a ‘*sticky floor*’ in healthcare contexts have been confirmed as metaphors that intertwine with a less visible but equally incisive phenomenon: the systemic advantage that men, albeit a minority in nursing, enjoy. This structural imbalance confirms Social Role Theory (SRT), emphasising that the organisational context is not neutral but produces and reproduces gender hierarchies (Eagly and Wood 1999). At an international policy level, the International Council of Nurses (2023) also emphasises that addressing gender inequities in the health and nursing workforce is essential for building sustainable and equitable health systems.

The combination of low subjective motivation and limited objective opportunities contributes to the crystallisation of a system in which female nursing leadership remains exceptional rather than structural. Some studies propose corrective strategies such as structured mentoring, flexibility policies and empowerment pathways, but the effectiveness of such strategies still appears marginal unless accompanied by a more profound cultural change (Gauci et al. 2023; Harris et al. 2021; Schwartz et al. 2021; Valentini and Di Marcantonio 2023).

The results of this review are part of a series of international studies that have long highlighted the persistence of systemic barriers to female leadership. Studies conducted in the healthcare sector confirm the lack of inclusive policies and the need to promote gender equality values in healthcare organisations, emphasising how ethical leadership can improve female inclusion in healthcare management and promote a culture of equal opportunities when aligned with institutional change processes (James et al. 2025; Mucheru et al. 2024; Pincha Baduge et al. 2023).

Nursing is a paradoxical context: despite being a predominantly female profession (Australian College of Nursing 2019), access to senior roles remains limited and highly asymmetrical, thus giving substance to the phenomena of the glass ceiling and glass elevator (Chisholm‐Burns et al. 2017; Kapoor et al. 2021; Newman 2014).

Compared to existing literature, this paper offers an original contribution both in terms of its specific focus on the nursing context and its multi‐level theoretical framework, which connects individual, cultural and organisational dimensions shaping women's leadership.

### 6.1 Limits

This review was conducted according to the REA method, which, while maintaining methodological rigour, is exploratory and does not guarantee the exhaustiveness of the available evidence, as would be the case in a systematic review. The methodological heterogeneity of the included studies made the comparative analysis more complex, while offering an integrated perspective on the phenomenon. While the number of included studies was limited, this reflects the REA's focus on methodological rigour and avoidance of overlapping evidence.

The generalizability of the results to different healthcare systems and cultural settings should be interpreted with caution. The proposed theoretical framework allowed for an integrated reading of the phenomena, but also involves a certain degree of abstraction. Some theoretical concepts, although relevant to the analysis, may not have been explicitly considered by the authors of the original studies, but were interpreted in the review process, with the epistemological limitations that this entails.

### 6.2 Implications and Recommendations for the Practice

The findings indicate that strategies to promote women's access to leadership roles in nursing should go beyond individual interventions and be embedded within transformative organisational and cultural policies. The promotion of female leadership requires both the development of strategic skills and the adoption of structural mechanisms for equity. Nurses may not have the right knowledge and specific skills regarding the impact of gender stereotypes; therefore, continuous professional development and training on the promotion of equal opportunities between women and men are recommended. From a managerial perspective, nursing leaders should implement structured mentoring programmes, transparent promotion criteria and regular gender‐sensitivity audits to identify and address organisational biases. Leadership development pathways tailored for women, such as shadowing senior managers or participating in strategic decision‐making committees, can foster empowerment and visibility.

Although few studies in the review directly evaluated the impact of interventions, several discussed strategies such as mentoring, bias‐awareness training and transparent promotion processes as promising practices for advancing gender equity in nursing leadership (Doleman et al. 2024; Mousa et al. 2023; Pincha Baduge et al. 2024; Saville et al. 2024). Future research should build on these insights by assessing the effectiveness and long‐term outcomes of such initiatives.

In clinical settings, inclusive leadership models that value relational and collaborative competencies should be integrated into team management practices. Encouraging distributed leadership within nursing teams can also strengthen professional autonomy and challenge hierarchical norms traditionally aligned with male leadership styles. This awareness could also be extended to future nurses during their academic studies to promote early awareness of the issue and break down unconscious prejudices in the profession that can hinder career expectations from the outset.

These implications are particularly relevant in the Italian context, where women represent approximately 76% of the nursing workforce yet remain underrepresented in formally recognised leadership positions. Recent national data indicate a leadership index of 1.4, signalling male prevalence in managerial roles despite men comprising only a minority of the workforce (D'Artibale et al. 2025). This imbalance reflects persistent structural and cultural barriers, and current projections suggest that achieving gender equity in nursing leadership may still require several decades of sustained organisational and cultural change (International Council of Nurses 2023; World Health Organization 2022).

However, the available literature also highlights the need for further specific empirical studies, both internationally and within the Italian context, to investigate the local dynamics that contribute to the persistence of inequalities. Future research should adopt larger, multi‐site and longitudinal designs to explore how organisational and cultural factors shape leadership trajectories over time. Comparative and qualitative studies could further elucidate cross‐national variations and the lived experiences of women in leadership, providing evidence to guide equity‐oriented health policies and management practices.

Ultimately, achieving gender equity in nursing leadership requires sustained political commitment, organisational accountability and the systematic inclusion of gender‐sensitive indicators in healthcare governance. Only through this multi‐layered approach can nursing leadership evolve from exception to norm within an equitable and inclusive professional culture.

## 7 Conclusion

This review offers a solid and innovative contribution to understanding the systemic barriers that limit female leadership in the nursing profession, providing a theoretical and analytical basis useful for future research and policy actions. The integrated analysis of data and theoretical references highlights that barriers to female nursing leadership are the result of a complex system of cultural norms, implicit biases, institutional mechanisms and internalised perceptions. These barriers are systematically intertwined, leading to unequal career paths, silent exclusion and under‐representation in decision‐making roles. To effectively address this phenomenon, interventions aimed solely at individual development are not sufficient. It is necessary to adopt multi‐level strategies capable of acting simultaneously on the individual, relational and structural levels. The picture that emerges calls on healthcare institutions, nursing contexts and training agencies to take an active role in transforming organisational cultures and power mechanisms, promoting inclusive, transparent environments capable of enhancing the potential of women in senior roles.

Only conscious, systemic and contextualised change can truly contribute to the creation of more equitable, representative and sustainable healthcare leadership. This rapid assessment of the evidence provides a basis for future targeted investigations and the development of evidence‐based interventions aimed at promoting gender equality in nursing leadership, thereby strengthening organisational justice within healthcare systems.

## Funding

The authors have nothing to report.

## Conflicts of Interest

The authors declare no conflicts of interest.

## Supporting information


**Data S1:** jan70570‐sup‐0001‐Supinfo1.docx.

**Data S2:** jan70570‐sup‐0002‐Supinfo2.docx.

## Data Availability

Data supporting the findings of this study are available from the corresponding author on reasonable request.

## References

[jan70570-bib-0001] Ajzen, I. 1991. “The Theory of Planned Behavior.” Organizational Behavior and Human Decision Processes 50, no. 2: 179–211.

[jan70570-bib-0002] Alghamdi, M. G. , R. Topp , and M. S. AlYami . 2018. “The Effect of Gender on Transformational Leadership and Job Satisfaction Among Saudi Nurses.” Journal of Advanced Nursing 74, no. 1: 119–127. 10.1111/jan.13385. 28714146

[jan70570-bib-0003] Aspinall, C. , S. Jacobs , and R. Frey . 2023. “Intersectionality and Nursing Leadership: An Integrative Review.” Journal of Clinical Nursing 32, no. 11–12: 2466–2480. 10.1111/jocn.16347. 35579183

[jan70570-bib-0004] Australian College of Nursing . 2019. Men in Nursing. Australian College of Nursing. https://www.acn.edu.au/men‐in‐nursing.

[jan70570-bib-0005] Bandura, A. 1977. “Self‐Efficacy: Toward a Unifying Theory of Behavioral Change.” Psychological Review 84, no. 2: 191–215. 847061 10.1037//0033-295x.84.2.191

[jan70570-bib-0006] Bettany‐Saltikov, J. , and R. McSherry . 2024. How to Do a Systematic Literature Review in Nursing: A Step‐By‐Step Guide. 3rd ed. Open University Press.

[jan70570-bib-0007] Beugelsdijk, S. , M. J. Gelfand , and J. Kleinhempel . 2025. “Evolving Patterns of Gender Inequality Over Time and Across Countries: New Theoretical Perspectives and an Emerging Research Agenda.” Journal of International Business Studies. 10.1057/s41267-025-00799-7.

[jan70570-bib-0008] Brown, C. , and L. Jones . 2004. “The Gender Structure of the Nursing Hierarchy: The Role of Human Capital.” Gender, Work and Organization 11, no. 1: 1–25. 10.1111/j.1468-0432.2004.00218.x.

[jan70570-bib-0009] Cardiff, S. , B. McCormack , and T. McCance . 2018. “Person‐Centred Leadership: A Relational Approach to Leadership Derived Through Action Research.” Journal of Clinical Nursing 27, no. 15–16: 3056–3069. 29679402 10.1111/jocn.14492

[jan70570-bib-0010] Chisholm‐Burns, M. A. , C. A. Spivey , T. Hagemann , and M. A. Josephson . 2017. “Women in Leadership and the Bewildering Glass Ceiling.” American Journal of Health‐System Pharmacy 74, no. 5: 312–324. 10.2146/ajhp160930. 28122701

[jan70570-bib-0011] Clayton‐Hathway, K. , A. L. Humbert , S. Schutz , R. McIlroy , and H. Griffiths . 2020. Gender and Nursing as a Profession: Valuing Nurses and Paying Them Their Worth. Royal College of Nursing.

[jan70570-bib-0012] Cottingham, M. D. 2019. “The Missing and Needed Male Nurse: Discursive Hybridization in Professional Nursing Texts.” Gender, Work and Organization 26, no. 2: 197–213. 10.1111/gwao.12333.

[jan70570-bib-0013] D'Artibale, M. , M. Marsilio , A. Prenestini , A. Poli , and L. Magnani . 2025. “Osservatorio Sull'equità di Genere Della Leadership in Sanità: Rapporto Annuale 2025 (Working Paper).” *Zenodo*. 10.5281/zenodo.17433410.

[jan70570-bib-0014] Doleman, G. , C. Duffield , and I. W. Li . 2024. “The Gender Pay Gap in the Australian Nursing Workforce: A Retrospective Observational Study.” Collegian 31, no. 6: 375–381. 10.1016/j.colegn.2024.09.002.

[jan70570-bib-0015] Eagly, A. H. , and A. Mladinic . 1994. “Are People Prejudiced Against Women? Some Answers From Research on Attitudes, Gender Stereotypes, and Judgments of Competence.” European Review of Social Psychology 5, no. 1: 1–35.

[jan70570-bib-0016] Eagly, A. H. , and V. J. Steffen . 1984. “Gender Stereotypes Stem From the Distribution of Women and Men Into Social Roles.” Journal of Personality and Social Psychology 46, no. 4: 735–754.

[jan70570-bib-0017] Eagly, A. H. , and W. Wood . 1999. “The Origins of Sex Differences in Human Behavior: Evolved Dispositions Versus Social Roles.” American Psychologist 54, no. 6: 408–423. 10.1037/0003-066X.54.6.408.

[jan70570-bib-0018] Evans, J. 2004. “Men Nurses: A Historical and Feminist Perspective.” Journal of Advanced Nursing 47, no. 3: 321–328. 10.1111/j.1365-2648.2004.03096.x. 15238127

[jan70570-bib-0019] Fiske, S. T. 2000. “Stereotyping, Prejudice, and Discrimination at the Seam Between the Centuries: Evolution, Culture, Mind, and Brain.” European Journal of Social Psychology 30, no. 3: 299–322. 10.1002/(SICI)1099-0992(200005/06)30:3<299::AID-EJSP2>3.0.CO;2-F.

[jan70570-bib-0020] FitzGerald, C. , and S. Hurst . 2017. “Implicit Bias in Healthcare Professionals: A Systematic Review.” BMC Medical Ethics 18, no. 1: 19. 10.1186/s12910-017-0179-8. 28249596 PMC5333436

[jan70570-bib-0021] Frantzen, K. K. , and M. D. Fetters . 2016. “Meta‐Integration for Synthesizing Data in a Systematic Mixed Studies Review: Insights From Research on Autism Spectrum Disorder.” Quality & Quantity 50, no. 5: 2251–2277. 10.1007/s11135-015-0261-6.

[jan70570-bib-0022] Gauci, P. , L. Luck , K. O'Reilly , and K. Peters . 2023. “Workplace Gender Discrimination in the Nursing Workforce—An Integrative Review.” Journal of Clinical Nursing 32, no. 17–18: 5693–5711. 10.1111/jocn.16684. 36922724

[jan70570-bib-0023] Glick, P. , and S. T. Fiske . 2018. “The Ambivalent Sexism Inventory: Differentiating Hostile and Benevolent Sexism.” In Social Cognition, 116–160. Routledge.

[jan70570-bib-0024] Global Health 50/50 . 2020. “Power, Privilege and Priorities.” Global Health 50/50 Report. https://globalhealth5050.org/wp‐content/uploads/2020/03/Power‐Privilege‐and‐Priorities‐2020‐Global‐Health‐5050‐Report.pdf.

[jan70570-bib-0025] Goldsmith, M. R. , C. R. Bankhead , and J. Austoker . 2007. “Synthesising Quantitative and Qualitative Research in Evidence‐Based Patient Information.” Journal of Epidemiology & Community Health 61, no. 3: 262–270. 10.1136/jech.2006.046110. 17325406 PMC2652927

[jan70570-bib-0026] Granikov, V. , Q. N. Hong , and P. Pluye . 2022. “Mixing Qualitative and Quantitative Evidence in a Systematic Review: Methodological Guidance With a Worked Example of Collaborative Information Monitoring.” In Handbook of Research on Mixed Methods Research in Information Science, 125–146. IGI Global Scientific Publishing. 10.4018/978-1-7998-8844-4.ch007.

[jan70570-bib-0027] Greene, J. , M. M. El‐Banna , L. A. Briggs , and J. Park . 2017. “Gender Differences in Nurse Practitioner Salaries.” Journal of the American Association of Nurse Practitioners 29, no. 11: 667–672. 10.1002/2327-6924.12512. 28857491

[jan70570-bib-0028] Hannawi, S. , and I. Al Salmi . 2018. “Time to Address Gender Inequalities Against Female Physicians.” International Journal of Health Planning and Management 33, no. 2: 532–541. 10.1002/hpm.2476. 29124785

[jan70570-bib-0029] Harris, C. E. , S. D. Clark , S. S. Chesak , et al. 2021. “GRIT: Women in Medicine Leadership Conference Participants' Perceptions of Gender Discrimination, Disparity, and Mitigation.” Mayo Clinic Proceedings: Innovations, Quality & Outcomes 5, no. 3: 548–559. 10.1016/j.mayocpiqo.2021.02.007. PMC8240154 34195547

[jan70570-bib-0030] Harrison, R. , B. Jones , P. Gardner , and R. Lawton . 2021. “Quality Assessment With Diverse Studies (QuADS): An Appraisal Tool for Methodological and Reporting Quality in Systematic Reviews of Mixed‐Or Multi‐Method Studies.” BMC Health Services Research 21, no. 1: 144. 10.1186/s12913-021-06122-y. 33588842 PMC7885606

[jan70570-bib-0031] Heilman, M. E. 2001. “Description and Prescription: How Gender Stereotypes Prevent Women's Ascent Up the Organizational Ladder.” Journal of Social Issues 57, no. 4: 657–674. 10.1111/0022-4537.00234.

[jan70570-bib-0032] Heise, L. , M. E. Greene , N. Opper , et al. 2019. “Gender Inequality and Restrictive Gender Norms: Framing the Challenges to Health.” Lancet 393, no. 10189: 2440–2454. 10.1016/S0140-6736(19)30652-X. 31155275

[jan70570-bib-0033] International Council of Nurses . 2023. Gender Equity in the Nursing and Health Workforce. International Council of Nurses. https://www.icn.ch/sites/default/files/2024‐01/PS_Gender%20equity%20in%20the%20nursing%20and%20healthcare%20workforce%20%20FINAL%2012.2023_EN.pdf.

[jan70570-bib-0034] International Labour Organization . 2022. “Le Lavoratrici Del Settore Sanitario e Della Cura Guadagnano il 24 Per Cento in Meno Rispetto al Personale di Sesso Maschile.” https://www.ilo.org/it/resource/news/le‐lavoratrici‐del‐settore‐sanitario‐e‐della‐cura‐guadagnano‐il‐24‐cento.

[jan70570-bib-0035] James, Y. , B. J. Hermosura , R. Decady , and I. L. Bourgeault . 2025. “Gender and Healthcare Leadership: Addressing Critical Knowledge Gaps by Explicitly Considering the Gendered Concept of Care.” Healthcare Management Forum 38, no. 2: 141–147. 39489512 10.1177/08404704241293947 PMC11849242

[jan70570-bib-0036] Jarman, J. , R. M. Blackburn , B. Brooks , and E. Dermott . 1999. “Gender Differences at Work: International Variations in Occupational Segregation.” Sociological Research Online 4, no. 1: 29–50. 10.5153/sro.206.

[jan70570-bib-0037] Joanna Briggs Institute . 2022. Checklist for Analytical Cross‐Sectional Studies. JBI.

[jan70570-bib-0038] Kapoor, D. , T. Sardana , and D. Sharma . 2021. “Women as Leaders: A Systematic Review of Glass Ceiling and Organisational Development.” International Journal of Indian Psychology 9, no. 1: 572–591. 10.25215/0901.058.

[jan70570-bib-0039] Kark, R. , and C. Buengeler . 2024. “Wo∼Men and Leadership: Re‐Thinking the State of Research on Gender and Leadership Through Waves of Feminist Thinking.” Journal of Leadership and Organizational Studies 31, no. 3: 245–266. 10.1177/15480518241257105.

[jan70570-bib-0040] Kelly, E. H. , T. Miskimen , F. Rivera , L. E. Peterson , and S. T. Hingle . 2021. “Women's Wellness Through Equity and Leadership (WEL): A Program Evaluation.” Pediatrics 148, no. Supplement 2: e2021051440I. 10.1542/peds.2021-051440I. 34470884

[jan70570-bib-0041] Khangura, S. , K. Konnyu , R. Cushman , J. Grimshaw , and D. Moher . 2012. “Evidence Summaries: The Evolution of a Rapid Review Approach.” Systematic Reviews 1: 10. 10.1186/2046-4053-1-10. 22587960 PMC3351736

[jan70570-bib-0042] Lane, N. , and N. F. Piercy . 2003. “The Ethics of Discrimination: Organizational Mindsets and Female Employment Disadvantage.” Journal of Business Ethics 44, no. 4: 313–325. 10.1023/A:1023644602447.

[jan70570-bib-0043] Lizarondo, L. , C. Stern , S. Salmond , et al. 2025. “Methods for Data Extraction and Data Transformation in Convergent Integrated Mixed Methods Systematic Reviews.” JBI Evidence Synthesis 23, no. 3: 429–440. 10.11124/JBIES-24-00331. 39829248

[jan70570-bib-0044] Llorens, A. , A. Tzovara , L. Bellier , et al. 2021. “Gender Bias in Academia: A Lifetime Problem That Needs Solutions.” Neuron 109, no. 13: 2047–2074. 34237278 10.1016/j.neuron.2021.06.002 PMC8553227

[jan70570-bib-0045] McMurry, T. B. 2011. “The Image of Male Nurses and Nursing Leadership Mobility.” Nursing Forum 46, no. 1: 22–28. 10.1111/j.1744-6198.2010.00206.x. 21306392

[jan70570-bib-0046] Mousa, M. , B. Garth , J. A. Boyle , K. Riach , and H. J. Teede . 2023. “Experiences of Organizational Practices That Advance Women in Health Care Leadership.” JAMA Network Open 6, no. 3: e233532. 10.1001/jamanetworkopen.2023.3532. 36939704 PMC10028487

[jan70570-bib-0047] Mucheru, D. , E. McAuliffe , A. Kesale , and B. Gilmore . 2024. “A Rapid Realist Review on Leadership and Career Advancement Interventions for Women in Healthcare.” BMC Health Services Research 24: 856. 10.1186/s12913-024-11348-7. 39069605 PMC11285393

[jan70570-bib-0048] Muench, U. , and H. Dietrich . 2019. “The Male‐Female Earnings Gap for Nurses in Germany: A Pooled Cross‐Sectional Study of the Years 2006 and 2012.” International Journal of Nursing Studies 89: 125–13110.1016/j.ijnurstu.2017.07.006. 28716298 10.1016/j.ijnurstu.2017.07.006

[jan70570-bib-0049] Newman, C. 2014. “Time to Address Gender Discrimination and Inequality in the Health Workforce.” Human Resources for Health 12, no. 1: 25. 10.1186/1478-4491-12-25. 24885565 PMC4014750

[jan70570-bib-0050] Odei, B. C. , P. Gawu , S. Bae , et al. 2021. “Evaluation of Progress Toward Gender Equity Among Departmental Chairs in Academic Medicine.” JAMA Internal Medicine 181, no. 4: 548–550. 10.1001/jamainternmed.2020.6267. 33369632 PMC7770616

[jan70570-bib-0051] Page, M. J. , J. E. McKenzie , P. M. Bossuyt , et al. 2021. “The PRISMA 2020 Statement: An Updated Guideline for Reporting Systematic Reviews.” BMJ 372: n71. 10.1136/bmj.n71. 33782057 PMC8005924

[jan70570-bib-0052] Pincha Baduge, M. d. S. , B. Garth , L. Boyd , et al. 2024. “Barriers to Advancing Women Nurses in Healthcare Leadership: A Systematic Review and Meta‐Synthesis.” EClinicalMedicine 67: 102354. 10.1016/j.eclinm.2023.102354. 38314055 PMC10837541

[jan70570-bib-0053] Pincha Baduge, M. S. D. S. , M. Mousa , B. Garth , L. Boyd , and H. J. Teede . 2023. “Organisational Strategies for Women Nurses to Advance in Healthcare Leadership: A Systematic Review.” Journal of Nursing Management 2023, no. 1: 2678916. 40225680 10.1155/2023/2678916 PMC11918808

[jan70570-bib-0054] Reynolds, R. , D. Rogers , L. Crawley , H. L. Richards , and D. G. Fortune . 2022. “Glass Ceiling Versus Sticky Floor, Sideways Sexism and Priming a Manager to Think Male as Barriers to Equality in Clinical Psychology: An Interpretive Phenomenological Analysis Approach.” Professional Psychology: Research and Practice 53, no. 3: 225–233. 10.1037/pro0000456.

[jan70570-bib-0055] Royal College of Nursing . 2020. RCN UK Labour Market Review 2020: A Report on the Present Position and Future Trends for Nursing. Royal College of Nursing. https://www.rcn.org.uk/‐/media/royal‐college‐of‐nursing/documents/publications/2021/march/009‐579.pdf.

[jan70570-bib-0056] Saville, N. M. , R. Uppal , S. A. Odunga , et al. 2024. “Pathways to Leadership: What Accounts for Women's (In)equitable Career Paths in the Health Sectors in India and Kenya? A Scoping Review.” BMJ Global Health 2024: e014745. 10.1136/bmjgh-2023-014745. PMC11261739 39019545

[jan70570-bib-0057] Schwartz, J. M. , S. D. Markowitz , S. D. Yanofsky , et al. 2021. “Empowering Women as Leaders in Pediatric Anesthesiology: Methodology, Lessons, and Early Outcomes of a National Initiative.” Anesthesia & Analgesia 133, no. 6: 1497–1509. 10.1213/ANE.0000000000005740. 34517375

[jan70570-bib-0058] Smith, S. G. , and J. C. Sinkford . 2022. “Gender Equality in the 21st Century: Overcoming Barriers to Women's Leadership in Global Health.” Journal of Dental Education 86, no. 9: 1144–1173. 10.1002/jdd.13059. 36165260

[jan70570-bib-0059] Srivastava, N. , and R. Nalawade . 2023. “Glass Ceiling to Sticky Floor: Analogies of Women Leadership.” International Journal of Professional Business Review 8, no. 4: 20.

[jan70570-bib-0060] Stanley, D. , and K. Stanley . 2018. “Clinical Leadership and Nursing Explored: A Literature Search.” Journal of Clinical Nursing 27, no. 9–10: 1730–1743. 29076264 10.1111/jocn.14145

[jan70570-bib-0061] Stern, C. , L. Lizarondo , J. Carrier , et al. 2020. “Methodological Guidance for the Conduct of Mixed Methods Systematic Reviews.” JBI Evidence Synthesis 18, no. 10: 2108–2118. 10.11124/JBISRIR-D-19-00169. 32813460

[jan70570-bib-0062] Tirumalai, A. A. , E. L. George , A. Kashikar , et al. 2021. “Gender Disparity in Surgical Society Leadership and Annual Meeting Programs.” Journal of Surgical Research 266: 69–76. 10.1016/j.jss.2021.02.023. 33984733

[jan70570-bib-0063] Turkmen, B. , and F. E. Bacaksiz . 2021. “Does the Glass Elevator Still Work: A Descriptive and Cross‐Sectional Study in the Context of Gender in Turkey.” Journal of Nursing Management 29, no. 5: 1275–1283. 10.1111/jonm.13266. 33484591

[jan70570-bib-0064] Valentini, I. , and M. Di Marcantonio . 2023. “Etica e Leadership Nel Sistema Sanitario Italiano: Risultati di Una Ricerca Empirica.” Mecosan: Management Ed Economia Sanitaria 126, no. 1: 29–46.

[jan70570-bib-0065] Varker, T. , D. Forbes , L. Dell , et al. 2015. “Rapid Evidence Assessment: Increasing the Transparency of an Emerging Methodology.” Journal of Evaluation in Clinical Practice 21, no. 6: 1199–1204. 10.1111/jep.12405. 26123092

[jan70570-bib-0066] Watt, A. , A. Cameron , L. Sturm , et al. 2008. “Rapid Reviews Versus Full Systematic Reviews: An Inventory of Current Methods and Practice in Health Technology Assessment.” International Journal of Technology Assessment in Health Care 24, no. 2: 133–139. 10.1017/S0266462308080185. 18400114

[jan70570-bib-0067] Whittemore, R. , and K. Knafl . 2005. “The Integrative Review: Updated Methodology.” Journal of Advanced Nursing 52, no. 5: 546–553. 16268861 10.1111/j.1365-2648.2005.03621.x

[jan70570-bib-0068] Williams, C. L. 2013. “The Glass Escalator, Revisited: Gender Inequality in Neoliberal Times, SWS Feminist Lecturer.” Gender & Society 27, no. 5: 609–629. 10.1177/0891243213490232.

[jan70570-bib-0069] World Health Organization . 2019. Delivered by Women, Led by Men: A Gender and Equity Analysis of the Global Health and Social Workforce. Human Resources for Health Observer Series N. 24. https://www.who.int/publications/i/item/9789241515467.

[jan70570-bib-0070] World Health Organization . 2021. “Closing the Leadership Gap: Gender Equity and Leadership in the Global Health and Care Workforce.” https://www.who.int/publications/i/item/9789240025905.

[jan70570-bib-0071] World Health Organization . 2022. “The Gender Pay Gap in the Health and Care Sector: A Global Analysis in the Time of COVID‐19.” https://www.who.int/publications/i/item/9789240052895.

