# Supplementary material for: Barriers to Women's Leadership in Nursing: A Rapid Evidence Assessment and Proposal for an Integrated Theoretical Framework
Source: J Adv Nurs. 2026 Apr 10;82(10):8956–67. doi: 10.1111/jan.70570 (PMC13576831; doi:10.1111/jan.70570)
Supplement: Supplementary file 1 [file JAN-82-8956-s001.docx]

**TABLE S1:** Quality appraisal for diverse studies (QuADS) (Harrison et al., 2021)

| **QuADS Criteria** | **Aspinall et al. (2023)** | **Pincha Baduge et al. (2024)** | **Gauci et al. (2023)** | **Saville et al. (2024)** | **Mousa et al. (2023)** |
| --- | --- | --- | --- | --- | --- |
| ***Theoretical or conceptual underpinning to the research*** | **3** | **3** | **2** | **3** | **3** |
| ***Statement of research aim/s*** | **3** | **3** | **2** | **3** | **3** |
| ***Clear description of research setting and target population*** | **3** | **3** | **3** | **3** | **3** |
| ***The study design is appropriate to address the stated research aim/s*** | **2** | **2** | **1** | **2** | **2** |
| ***Appropriate sampling to address the research aim/s*** | **2** | **3** | **2** | **3** | **3** |
| ***Rationale for choice of data collection tool/s*** | **3** | **3** | **2** | **3** | **3** |
| ***The format and content of data collection tool is appropriate to address the stated research aim/s*** | **2** | **2** | **1** | **3** | **2** |
| ***Description of data collection procedure*** | **3** | **3** | **2** | **2** | **3** |
| ***Recruitment data provided*** | **3** | **3** | **2** | **2** | **3** |
| ***Justification for analytic method selected*** | **2** | **3** | **1** | **2** | **3** |
| ***The method of analysis was appropriate to answer the research aim/s*** | **2** | **2** | **1** | **2** | **2** |
| ***Evidence that the research stakeholders have been considered in research design or conduct*** | **3** | **3** | **2** | **3** | **3** |
| ***Strengths and limitations critically discussed*** | **2** | **3** | **2** | **2** | **3** |
| **Total score** | ***32*** | ***36*** | ***23*** | ***33*** | ***36*** |

**TABLE S2:** JBI Critical Appraisal Checklist for Analytical Cross-Sectional Studies (JBI,2022)

| **JBI Criteria** | **Greene et al. (2017)** | **Doleman et al. (2024)** | **Muench&Dietrich (2019)** |
| --- | --- | --- | --- |
| ***Were the criteria for inclusion in the sample clearly defined?*** | **Yes** | **Yes** | **Yes** |
| ***Were the study subjects and the setting described in detail?*** | **Yes** | **Yes** | **Yes** |
| ***Was the exposure measured in a valid and reliable way?*** | **Yes** | **Yes** | **Yes** |
| ***Were objective, standard criteria used for measurement of the condition?*** | **Yes** | **Yes** | **Yes** |
| ***Were confounding factors identified?*** | **Yes** | **Yes** | **No** |
| ***Were strategies to deal with confounding factors stated?*** | **Yes** | **Yes** | **No** |
| ***Were the outcomes measured in a valid and reliable way?*** | **Yes** | **Yes** | **Yes** |
| ***Was appropriate statistical analysis used?*** | **Yes** | **Yes** | **Yes** |
| **Total score** | ***High*** | ***High*** | ***Moderate*** |

**TABLE S3:** Data extraction literature reviews

| **Authors**  **(Year of publication)** | **Aim/Research focus** | **Geographic location (Country)** | **Sample Description** | **Review design** | **Main Results** | **Conclusions and implications for practice** |
| --- | --- | --- | --- | --- | --- | --- |
| Aspinall C. et al.  (2022) | To synthesize international evidence on how intersectionality can be used to analyze inequalities within the nursing profession, with a specific focus on leadership. | New Zealand | N= 16 studies (10 qualitative  2 quantitative, 4  mix methods) published between 2011 and 2021 selected by CINAHL Plus, PsycINFO, PubMed, Ovid, ProQuest and Google Scholar. | Integrative review using directed content analysis by a feminist intersectionality framework. | Data were coded using a theory framework centered on three intersecting dimensions: power, privilege, and oppression.   (1) Power: although women comprise 75% of the nursing workforce, men occupy a disproportionate number of leadership roles. (2) Privilege: class privilege interests with gender and race, benefiting some while marginalizing others. (3) Oppression: women face multilayered oppression, often shaped by patriarchal expectations, limited access to education, and childcare-related role conflict. | Patriarchal structures and gendered societal expectations continue to facilitate men’s advancement into nursing leadership. The intersection of gender and ethnicity was identified as a key barrier to career progression. Intra-group differences shaped by social identities and cultural norms can reinforce dynamics of privilege and oppression. The review highlights a significant gap in research adopting an intersectional lens in health leadership studies.  **Implications for practice**  The authors advocate for the use of intersectionality not only as a theoretical framework but also as a practical tool to challenge power structures and inform inclusive leadership development in healthcare |
| Pincha Baduge M.S. et al.  (2023) | To explore the barriers that prevent women nurses from advancing into leadership roles in healthcare and academia. | Australia (review team); studies included were conducted in multiple countries such as Australia, USA, UK, Canada. | N= 32 studies (18 qualitative, 11 quantitative, 3 mix methods) published between January 2000 and October 2023 selected by MEDLINE, EMCARE, EMBASE, PsycINFO, PubMed, and CINAHL PLUS (via EBSCO host). | Systematic review and meta-synthesis mapped to the AMO framework (Abilities, Motivation, Opportunities) | Barriers were classified into three AMO domains.   (1) Abilities: lack of accessible role models, leadership training, and orientation. (2) Motivation: gendered expectations, bias, and lack of work-life support. (3) Opportunities: structural, organizational, and policy level barriers limiting leadership access. | The study highlights systemic, professional, and cultural barriers to nurses’ advancement into leadership roles. Social norms positioning nursing ad inherently feminine and attributing subordinate societal roles to women are identified as structural determinants that hinder career progression.  **Implications for practice**  The authors call multilayered, system level strategies to address gender disparities in nursing leadership. These include leadership development programs, structured mentoring, workplace flexibility, organizational reform, and cultural transformation. Intersectional and equity focused approaches are proposed as essential for sustainable and inclusive leadership in global healthcare systems. |
| Gauci P. et al.  (2023) | To critically synthesize workplace gender discrimination experiences among nurses, with a particular focus on differences between men and women. | Australia (review team), studies included were conducted in multiple countries such as Australia, USA, UK. | N= 20 studies (17 qualitative, 3 quantitative) published between 2012 and 2022 selected by CINAHL, MEDLINE, SCOPUS and Cochrane Library. | Integrative review using thematic analysis as a described by braun and Clarke (2006) | Four major themes were identified.   (1) Career progression: male nurses benefit from a “glass escalator” that facilitates rapid advancement in a female dominated profession. (2) Career interruption: female nurses experience limited professional development opportunities following maternity leave. (3) Positioning of men in nursing: Male nurses often receive preferential treatment but are also expected to undertake physically demanding tasks and assume a “protector” role. (4) Positioning of women in nursing: female nurses are perceived as a subordinate to medical professionals and lack influence within interdisciplinary teams. | Gender discrimination in nursing affects men and women differently. Strategies to increase male representation in nursing should not unintentionally reinforce existing patriarchal advantages.  **Implications for practice**  The authors recommend gender sensitive leadership education to address power dynamics in nursing. Awareness of one’s positionality and leadership authority is essential to critically understand and address the systemic subordination of women within the healthcare workforce |
| Saville M.S. et al. (2024) | To capture evidence on enablers and barriers to improving equal opportunity and effective organizational interventions that can advance women’s leadership in India and Kenya’s health sectors. | India and Kenya | N= 26 studies  (India=15;  Kenya=11)  (7 mix methods  11 qualitative,  5 quantitative,  3 commentary papers)  Published between 2008 and 2020. 7 studies specifically focused on the nursing workforce | Integrative review using inductive thematic analysis | 14 themes of facilitators and barriers were identified across the two countries at individual/interpersonal, organizational, structural, and cross-cutting normative levels.   (1) The main individual/interpersonal factors enabling or hindering women’s career progression across both countries were family support, knowledge, skills and personal attributes, and material resources. (2) Organizational factors enabling or hindering women’s career progression were capacity building and networking, career progression and leadership opportunities, organizational policies, work culture and relationships, flexibility and work burden. (3) Structure factor that hinder women’s access to leadership role include: government policies, national and state institutions and, access to public infrastructure. (4) Cross-cutting gender normative factors were gendered occupational segregation, unpaid care work burden and, norms around masculinity. | Women in the health sectors of India and Kenya face multifaceted barriers to leadership advancement. Structural, socio cultural, and institutional constraints require targeted interventions such as anti-discriminations and anti-harassment programs, leadership development, flexible work arrangements, and enhanced interpersonal and family support systems.  **Implication for practice**  The finding underscore the necessity for culturally contextualized strategies to advance women’s leadership in healthcare systems embedded patriarchal norms. Multilevel and systemic actions are essential to promote equity in female health leadership. |

**TABLE S4:** Data extraction quantitative studies

| **Authors**  **(Year of publication)** | **Aim/Research**  **focus** | **Geographic location (Country)** | **Sample Description** | **Review design** | **Main Results** | **Conclusions and implications for practice** |
| --- | --- | --- | --- | --- | --- | --- |
| Muench U. & Dietrich H.  (2017) | To examine the gender earnings gap among nurses in Germany, in a highly regulated public sector setting with standardized pay scales, to assess whether disparities persist even when typical explanations like motivation or negotiation are less applicable. | Germany | Nationally representative sample of 828 registered nurses (N=411 in 2006, N=417 in 2012), aged 21-65, actively employed in nursing, draw from the BIBB/BAuA Employment Surveys. | Pooled cross-sectional design using 2006 and 2012 data. Multivariate ordinary least squares (OLS) regression models were applied to log monthly gross earnings, adjusting for demographic characteristics, human capital, employment factors, and survey year. | (1) In unadjusted models, male nurses earned 30% more than female nurses; after full adjustment, the gender pay gap remained at 9.3% (approximately €260/month). (2) Female nurses worked 5.6 fewer contracted hours per month on average and had 4 more years of nursing experience than male nurses. (3) Male nurses who left nursing earned 16% more than those still employed in nursing; female nurses who left earned 6% less than those who stayed. (4) No evidence was found that gender differences in earning were explained by motivation-related variables. | Despite Germany’s highly regulated public sector pay structures, a persistent gender earnings gap was observed among nurses. This disparity remained after adjusting for demographic, occupational, and human capital variables, suggesting that traditional explanations such as motivation or individual productivity are insufficient to account for the difference. Instead, the results point to structural factors, including better outside labor market opportunities for male nurses, as partial explanations for the observed gap.  **Implication for Practice** To reduce gender-based pay disparities in nursing, institutions should implement transparent compensation policies and conduct regular wage audits. Policy-makers should consider mechanisms to support equitable career advancement and monitor transitions out of the nursing profession, particularly among male nurses, to ensure fair compensation structures are maintained across the workforce. |
| Greene J. et al.  (2017) | To examine whether the gender gap in NP salaries persist after controlling for differences in work setting and demographic factors; to assess when the gap emerges based on years since NP graduation; and to determine whether the gap varies by clinical specialty. | United States | N=6.591 Nurse Practitioners working as NPs at least 35 hours per week | Cross-sectional observational study using secondary analysis of the 2012 National Sample Survey of Nurse Practitioners. Bivariate and multivariate regression analyses were conducted to examine the association between gender and NP annual salary, adjusting for demographic, work setting, and autonomy variables. | (1) Male NPs earned $12,859 more than female NPs after adjustment. (2) The gender gap was $7,405 among recent NP graduates (0-4 years) and increased over time. (3) Male NPs earned significantly more in all clinical specialty areas, with the largest gap in surgical specialties ($24,285). (4) Racial and ethnic minority NPs earned & 4,257 more than White NPs.   NPs with greater autonomy (own provider number, own patient panel) had higher salaries. | This study reveals a persistent and significant gender pay gap among nurse practitioners in the United State, with male NPs earning substantially more than female NPs, even after adjusting for education, clinical setting, and demographic variables. The disparity is evident from the early stages of professional practice and widens with years of experience. The gap exists across all clinical specialties, with most pronounced differences in surgical fields. These findings point to systemic inequities in compensation that cannot be fully explained by measurable job-related factors.  **Implications for Practice**  Nursing schools should ensure students develop negotiation skills. Healthcare organizations should reassess hiring processes and conduct pay equity assessment to identify and ameliorate pay inequality. |
| Doleman G. et al.  (2024) | To examine the extent of gender pay gap in the Australian nursing workforce and identify the factors contributing to this disparity over the first three years post-graduation. | Australia | N= 4.250 registered nurses (90% female, mean age 32; 10% male, mean age 35) who responded to national graduate surveys at 6 months and 3 years post-graduation, covering all Australian universities. | Retrospective observational study using national longitudinal survey data of nursing graduates across Australia. | (1) A 4% gender pay gap was observed 6 months post-graduation, increasing to 13% at 3 years, favoring male nurses. (2) Gender was also associated with overqualification status, with men more likely to report working in higher-paid shifts (evening/weekends).   These disparities persisted despite controlling for employment sector, working hours, and location. | A statistically significant gender pay gap exist among Australian nursing graduates, beginning early in the career and widening over time. The disparity persists even after adjusting for key variables such as employment sector, hours worked, and geographic location, suggesting underlying systemic and gendered factors.  **Implication for Practice**  Findings call for workforce strategies that address structural gender inequities in nursing. Specific attention is needed to monitor pay progression by gender and to inform policies that improve fairness in remuneration, particularly in the early stages of professional careers. |

**TABLE S5:** Data extraction qualitative study

| **Authors**  **(Year of publication)** | **Aim/research focus** | **Geographic location (Country)** | **Sample Description** | **Study design methodology** | **Analysis** | **Main Results** | **Conclusions and implications for practice** |
| --- | --- | --- | --- | --- | --- | --- | --- |
| Mousa M. et al.  (2023) | To explore women’s lived experiences in healthcare leadership roles and identify how organizational practices support their advancement into leadership. | Australia | N=28 women with a purpositive sampling (10 nurses, 9 allied health; 9 medical disciplines). Participants had held leadership position for more than five years. | Qualitative study employing a constructivist grounded theory approach. | Semi-structured online interviews were conducted by the firs author. All interview recordings were transcribed verbatim and deidentified. Data collection and analysis were conducted iteratively, with constant comparative analysis throughout, beginning with the first transcript. Coding categories were constantly compared, examined, defined, and refined, generating increasingly more abstract concepts. | Our interrelated elements were identified that create the necessary conditions for an organizational culture to advance women in health care leadership, including:  (1) identifying and actively addressing systemic barriers;  (2) challenging gendered assumptions and expectations of leadership behaviors;  (3) providing mentorship to tangibly shape women’s career opportunities;  (4) determining how these conditions contribute toward raising women’s credibility to enable women to internalize a leadership identity. | Nurse participants, though in leadership roles, reported persistent barriers due to medical hierarchy and limited institutional visibility. They emphasized the need for profession specific internal supports and accessible role models. Practices most consistently reported ad enabling career progression were those deeply embedded within the organizational culture.  **Implications for practice**  The study offers a conceptual model highlighting how practices, culture, and environmental factors interact to shape women’s advancement trajectories. |
